# The Development of Neuroendocrine Disturbances over Time: Longitudinal Findings in Patients after Traumatic Brain Injury and Subarachnoid Hemorrhage

**DOI:** 10.3390/ijms17010002

**Published:** 2015-12-22

**Authors:** Anna Kopczak, Carmen Krewer, Manfred Schneider, Ilonka Kreitschmann-Andermahr, Harald Jörn Schneider, Günter Karl Stalla

**Affiliations:** 1 Clinical Neuroendocrinology Group, Max Planck Institute of Psychiatry, Kraepelinstraße 2-10, Munich 80804, Germany; stalla@psych.mpg.de; 2 Schön Klinik Bad Aibling, Kolbermoorer Straße 72, Bad Aibling 83043, Germany; CKrewer@schoen-kliniken.de (C.K.); MSchneider@schoen-kliniken.de (M.S.); 3 Department of Neurosurgery, University of Duisburg-Essen, Hufelandstraße 55, Essen 45147, Germany; ilonka.kreitschmann@uk-essen.de; 4 Department of Endocrinology, Ludwig-Maximilians-University, Ziemssenstraße 1, Munich 80336, Germany; harald.schneider@med.uni-muenchen.de

**Keywords:** hypopituitarism, traumatic brain injury, subarachnoid hemorrhage, neuroendocrinology, posttraumatic hypopituitarism

## Abstract

Previous reports suggest that neuroendocrine disturbances in patients with traumatic brain injury (TBI) or aneurysmal subarachnoid hemorrhage (SAH) may still develop or resolve months or even years after the trauma. We investigated a cohort of *n* = 168 patients (81 patients after TBI and 87 patients after SAH) in whom hormone levels had been determined at various time points to assess the course and pattern of hormonal insufficiencies. Data were analyzed using three different criteria: (1) patients with lowered basal laboratory values; (2) patients with lowered basal laboratory values or the need for hormone replacement therapy; (3) diagnosis of the treating physician. The first hormonal assessment after a median time of three months after the injury showed lowered hormone laboratory test results in 35% of cases. Lowered testosterone (23.1% of male patients), lowered estradiol (14.3% of female patients) and lowered insulin-like growth factor I (IGF-I) values (12.1%) were most common. Using Criterion 2, a higher prevalence rate of 55.6% of cases was determined, which correlated well with the prevalence rate of 54% of cases using the physicians’ diagnosis as the criterion. Intraindividual changes (new onset insufficiency or recovery) were predominantly observed for the somatotropic axis (12.5%), the gonadotropic axis in women (11.1%) and the corticotropic axis (10.6%). Patients after TBI showed more often lowered IGF-I values at first testing, but normal values at follow-up (*p* < 0.0004). In general, most patients remained stable. Stable hormone results at follow-up were obtained in 78% (free thyroxine (fT4) values) to 94.6% (prolactin values).

## 1. Introduction

Neuroendocrine disturbances are common after brain damage [1,2]. In previous reports with repeated hormone measurements, it has been found that neuroendocrine disturbances may still develop or improve months or even years after traumatic brain injury (TBI). While it is more common for hormonal disturbances to resolve, in a few cases, new insufficiencies developed with a latency of 12 months after injury [3,4] or after three years [5]. Recently, this finding was confirmed in a cohort of 82 Greek patients. In this cohort, pituitary insufficiency resolved in 10 out of 82 patients, whereas hypopituitarism was newly diagnosed in 16 cases [6]. In contrast to previous reports, more patients developed a new insufficiency than recovered from posttraumatic hypopituitarism. However, in this study, the mean follow-up time of four years was longer than in previous reports.

Hypothalamo-pituitary dysfunction is not only observed after TBI, but also after aneurysmal subarachnoid hemorrhage (SAH) [1,7,8]. When patients were investigated in the very early acute phase, hypopituitarism was reported to be rare after a median follow-up time of 30 months [9]. However, recent data suggest that there might be an increase of hypopituitarism from 34% to 41% within a two-year follow-up period [10]. Similar to TBI, recovery from pituitary insufficiency, as well as new onset hypopituitarism is documented after one and three years following SAH [11]. Therefore, some authors conclude that follow-up testing is mandatory in these patients [4,11].

In the classic pathologies that lead to hypopituitarism, such as intra- and supra-sellar tumors, the return of pituitary function is very unlikely after a certain amount of time. In such patients, the necessity of long-term hormone replacement can be prognosticated. However, given the fact that neuroendocrine function in patients after SAH or TBI can be considered as fluctuating to a certain degree, the indication for hormone replacement must be checked on a regular basis in order to prevent under-, but also over-treatment. This is exemplified by case reports by Agha *et al.* [12] and Streetz-van der Werf *et al.* [13], which highlight the consequences of over- or under-treating patients with neuroendocrine damage after TBI.

Against this background, we analyzed our cohort of patients in the Structured Data Assessment of Hypopituitarism after TBI and SAH to assess the prevalence and time course of neuroendocrine disturbances across the documented follow-up period.

The Structured Data Assessment of Hypopituitarism after TBI and SAH database is a multi-center database of TBI and SAH patients in which detailed clinical endocrine and outcome information has been collected. It offers the possibility of the inclusion of several follow-up assessments in order to characterize patients’ long-term course after the injury.

## 2. Results

### 2.1. Subjects with Repeated Hormone Measurements

In the Structured Data Assessment after TBI and SAH, a follow-up was conducted in 242 out of 1242 patients (117 patients after TBI and 125 patients after SAH). Since 36 patients (22 after TBI and 14 after SAH) received more than one longitudinal evaluation, a total number of 310 follow-up study sheets (167 sheets in patients after TBI and 143 sheets in patients after SAH) were available.

After quality control, we included only cases in which hormonal assessment had been repeated; follow-up study sheets with completion of hormonal values, but without repeated measurements were not defined as longitudinal findings and therefore excluded. Since changes in pituitary function require time to become manifest in laboratory values, we excluded all cases with a time interval of <2 weeks between primary examination and follow-up. Further exclusion criteria are displayed in the supplementary Figure S1. Using these criteria, 217 follow-up study sheets (119 follow-up study sheets in *n* = 81 patients after TBI, as well as 98 follow-up study sheets in *n* = 87 patients after SAH) remained for further analyses.

All 168 patients were investigated at least twice. In 22 out of 168 patients, evaluation was conducted at least thrice and in eight patients at least four times. One single patient after TBI was followed up to nine times.

The study sheets contained amongst other data information on current medication and basal hormonal laboratory values (cortisol, free thyroxine (fT4), testosterone, estradiol, prolactin and insulin-like growth factor I (IGF-I)). Basal laboratory values were documented in 144 patients out of 168 patients. In addition, the treating physicians had been asked at the time of data entry to classify their patients as pituitary sufficient or insufficient with regard to the corticotropic, thyrotropic, gonadotropic and somatotropic axis. This decision was driven not only by the basal hormonal laboratory values, but also by the patient`s history and clinical symptoms and signs of hypopituitarism. All physicians were aware of the basal laboratory values of their patients, but they were able to utilize further diagnostic information. This physicians’ diagnosis was available in 113 patients.

### 2.2. Basal Laboratory Values

**Figure 1:**
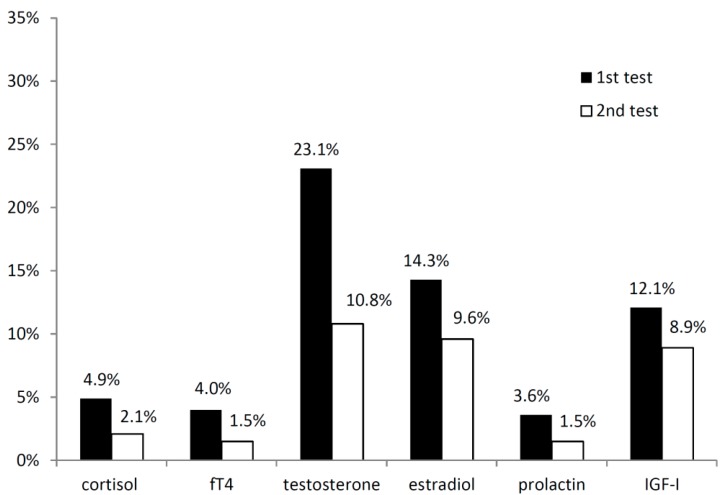
Lowered basal laboratory values in the first hormonal assessment after a median of three months (first test) and the follow-up after a median of seven months (second test) in patients after traumatic brain injury (TBI) and subarachnoid hemorrhage (SAH). Abbreviations: fT4, free thyroxine; IGF-I, insulin-like growth factor I.

In *n* = 123 patients, all neuroendocrine parameters (cortisol, fT4, testosterone or estradiol, respectively, prolactin and IGF-I) were documented at the first time of testing. In most patients (*n* = 80/123; 65.0%), the first hormonal assessment was normal. In 38 patients (30.9%), hormone parameters of a single pituitary axis were affected. *n* = 5 patients (4.1%) showed abnormal test results indicative of an impairment of two or more pituitary axes. The first hormonal assessment after a median time of three months after injury showed mainly low testosterone, estradiol and IGF-I values, possibly pointing towards gonadotropic and somatotropic insufficiencies (Figure 1). At follow-up after a median time of seven months after the injury, the prevalence rates of lowered hormonal values decreased (Figure 1). Especially in men, sex steroids often returned to normal, indicating a recovery of the respective hormone axis.

### 2.3. Patients on Hormone Replacement Therapy

Since our study has a naturalistic design, patients on hormone replacement therapy had also been included in the database. However, these patients had often normal basal laboratory values. Nevertheless, they suffered from pituitary insufficiency, which required a hormone replacement therapy. In order to have better insight into the prevalence of pituitary insufficiency after TBI and SAH, patients with lowered basal laboratory values, as well as patients requiring hormone replacement therapy with normal basal laboratory values were analyzed together and defined as “pituitary insufficient”.

Using this criterion, higher prevalence rates of disturbed hormonal axes indicative of pituitary insufficiency were observed. In most patients (44.4%), basal laboratory values were normal at the first hormonal assessment. In 61 patients (42.4%), a single pituitary axis was affected, which was indicated by a lowered basal laboratory value or hormone replacement therapy. *n* = 18 patients (12.5%) showed an impairment of two or more pituitary axes. One patient (0.7%) receiving hormone replacement therapy showed further lowered laboratory neuroendocrine parameters, thus indicating panhypopituitarism.

**Figure 2:**
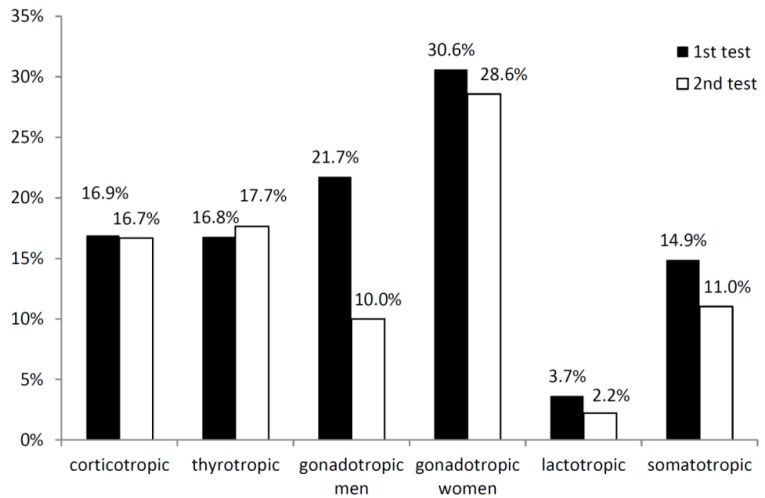
Pituitary insufficiencies defined by lowered basal laboratory values or receiving hormone replacement in the first hormonal assessment after a median of three months (first test) and the follow-up after a median of seven months (second test) in patients after traumatic brain injury (TBI) and subarachnoid hemorrhage (SAH).

The first hormonal assessment after a median time of three months after injury showed mainly results indicative of gonadotropic and of corticotropic insufficiencies (Figure 2). A follow-up after a median time of seven months after the injury revealed fewer results indicative of gonadotropic insufficiencies in men and a lower prevalence of assumed somatotropic and lactotropic insufficiencies. Interestingly, the prevalence rates of assumed corticotropic and thyrotropic insufficiencies remained stable (Figure 2).

Using pathological hormone levels or the need for hormone therapy as the criterion for pituitary insufficiency, prevalence rates did not differ significantly between TBI and SAH patients, except for lowered IGF-I values at follow-up. Lowered IGF-I values were observed in 26% in patients after TBI and in 8.3% in patients after SAH at follow-up (*p* = 0.007).

Moreover, intraindividual changes were observed after taking all follow-up tests into account and not only the second hormonal assessment. Most patients remained stable (insufficient or not) regarding the lactotropic axis (94.6%), which is clinically not so important. In other pituitary axes, stable results were mainly obtained in 77.8% to 84.5%. Because of the high percentage of unknown hormone replacement therapies with testosterone in men, data on this hormone in men cannot be compared to the other pituitary axes.

Hormone changes or changes in hormone replacement indicative of new onset insufficiency or recovery were predominantly observed in the somatotropic axis (12.5%) and in the corticotropic axis (10.6%). Further details are shown in Table 1.

**Table 1:** Intraindividual changes in pituitary insufficiencies defined by basal laboratory values or by the change of hormone replacement therapy between the first visit and the follow-up.

Impaired Hormonal Axes	Corticotropic		Thyrotropic		Gonadotropic		Somatotropic		Lactotropic	
Number of Patients	*n* = 123 (* *n* = 56)	%	*n* = 126 (* *n* = 60)	%	*n* = 118 (* *n* = 36)	%	*n* = 112 (* *n* = 52)	%	*n* = 111 (* *n* = 52)	%
Stable, no insufficiency	88	71.5	79	62.7	23	19.5	87	77.7	104	93.7
Stable, insufficiency	16	13.0	19	15.1	12	10.2	6	5.4	1	0.9
New insufficiency	10	8.1	7	5.6	2	1.7	5	4.5	2	1.8
Resolved	3	2.4	4	3.2	2	1.7	9	8.0	4	3.6
Unknown replacement	6	4.9	17	13.5	79	66.9	5	4.5	0	0.0

* Number of female patients.

In order to compare the group of patients with a stable result to the group of patients who showed changes in basal hormone measurements or hormone replacement therapy, we performed statistical analyses. Logistic regression analyses for the category of “change” in hormonal values/hormone replacement therapy were performed for each pituitary axis. The variables considered influential included “age at injury”, “sex”, “time between injury and first testing”, “type of injury” and “pituitary insufficiency at first visit”. There were no significant results except for the somatotropic axis in patients after TBI. Patients after TBI showed statistically more often lowered IGF-I values at first testing, but normal values at follow-up (*p* < 0.0004).

### 2.4. Physicians’ Diagnosis of Pituitary Insufficiency

In addition to the basal laboratory values, the treating physicians were asked to classify their patients as pituitary insufficient or not. This information was available in 113 patients. According to the physicians’ diagnosis, 52 patients (46%) showed no hormonal insufficiency. In 45 patients (39.8%), a single pituitary axis was disturbed. Two or more pituitary insufficiencies were documented in 15 patients (13.3%). The aforementioned patient in whom laboratory test results were severely abnormal and who was on hormone replacement therapy was also classified as having panhypopituitarism by the treating physician (0.9%).

**Figure 3:**
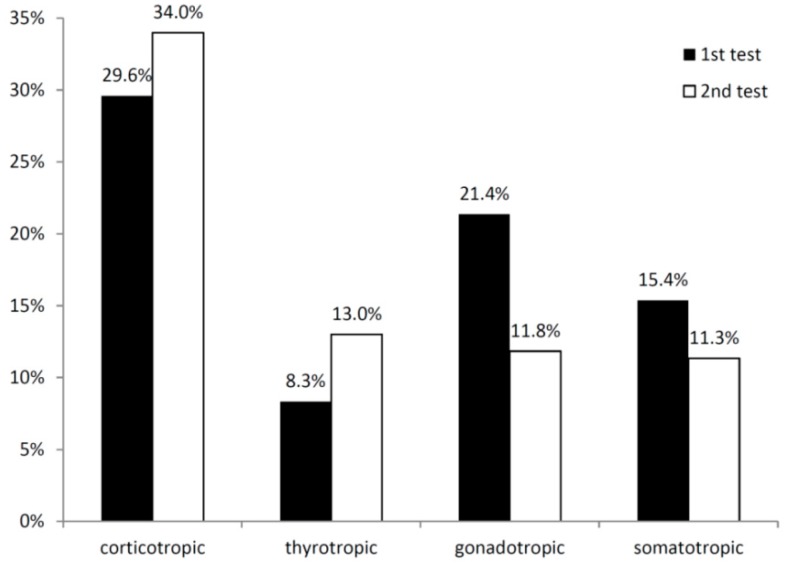
Pituitary insufficiencies defined by physicians’ diagnosis in the first hormonal assessment after a median of three months (first test) and the follow-up after a median of seven months (second test).

At the first visit, the most common pituitary insufficiency was corticotropic insufficiency (29.6%) followed by gonadotropic insufficiency (21.4%) (Figure 3). At follow-up, the prevalence of corticotropic and thyrotropic insufficiencies increased up to 34% or 13%, respectively, whereas the prevalence of gonadotropic and somatotropic insufficiencies decreased (Figure 3).

Intraindividual changes are displayed in Table 2. The thyrotropic axis was the most stable (94% without change), followed by the somatotropic axis (86.6%), the gonadotropic axis (84.5%) and the corticotropic axis (77.3%). Most insufficiencies resolved in the gonadotropic axis (11.9%). New onset insufficiencies were most common in the corticotropic axis (13.4%).

**Table 2:** Intraindividual changes in pituitary insufficiencies defined by the physicians’ diagnosis between the first visit and the follow-up.

Impaired Hormonal Axes	Corticotropic		Thyrotropic		Gonadotropic		Somatotropic	
Number of Patients	*n* = 97 (* *n* = 47)	%	*n* = 92 (* *n* = 45)	%	*n* = 84 (* *n* = 37)	%	*n* = 90 (* *n* = 45)	%
Stable, no insufficiency	55	56.7	80	87.0	63	75.0	74	82.2
Stable, insufficiency	20	20.6	7	7.6	8	9.5	4	4.4
New insufficiency	13	13.4	4	4.3	3	3.6	5	5.6
Resolved	9	9.3	1	1.1	10	11.9	7	7.8

* Number of female patients.

## 3. Discussion

In this study, we evaluated longitudinal findings in the Structured Data Assessment of Hypopituitarism after TBI and SAH. Follow-up evaluation of hypopituitarism was available in a large cohort of 168 patients with TBI and SAH.

In general, there are different ways to classify patients as hormonally insufficient. Basal laboratory values as the criterion can provide first insights into the function of the pituitary gland and are easy to obtain. In our cohort, 35% of patients showed lowered basal laboratory values at the first assessment, indicating a potential dysfunction of the respective hormone axis. Lowered sex hormone indicative of gonadotropic insufficiency and lowered IGF-I values indicative of somatotropic insufficiency were most common. These results are in line with previous reports [1,3].

It is well known that the prevalence rates of posttraumatic hypopituitarism differ from study to study due to different test methods and reference values [14]. In order to have better insight into the prevalence of hypopituitarism, we established further criteria of “pituitary insufficiency” in our study.

Since our study has a naturalistic design, patients on hormone replacement therapy had also been included. They often showed normal laboratory values due to the fact that a hormone replacement had been established. When patients on hormone replacement therapy had also been classified as pituitary insufficient independent of their (mostly normal) laboratory values, a higher prevalence rate of 55.6% was observed.

All patients were treated by experts in this research field in specialized centers. When the criterion “physicians’ diagnosis of hormonal insufficiency” was applied, nearly the same prevalence of posttraumatic hypopituitarism (54%) was observed. This demonstrates a good accordance between the purely mechanistic evaluation of neuroendocrine laboratory values after cerebral injury and the more holistic clinical and biochemical diagnosis of pituitary insufficiency by the treating doctor. Nevertheless, there was some degree of discrepancy. Regarding the different pituitary axes, lowered sexual hormones indicative of gonadotropic insufficiency were most common followed by lowered IGF-I values indicative of somatotropic insufficiency when basal laboratory values were applied as the criterion for pituitary insufficiency. Similar to this, gonadotropic insufficiency could be assumed to be the most common disorder using Criterion 2 (lowered basal hormonal values or hormone replacement therapy). However, when the physicians’ diagnosis was applied as the criterion, corticotropic insufficiency was the most frequently-diagnosed disorder followed by gonadotropic insufficiency. This result emphasizes that lowered laboratory values cannot be equalized automatically with the diagnosis of pituitary insufficiency, as it shown for the gonadotropic axis in this report, as well as in previous studies [15]. In our study, we believe the discrepancy between the criteria to be due to additional information available to the treating physicians from dynamic endocrine testing. In fact, treatment guidelines recommend dynamic testing for the evaluation of the corticotropic and somatotropic axes [2,16]. Recently, Klose *et al.* [17] reported that the diagnosis of growth hormone deficiency is highly dependent on growth hormone testing and confirmatory testing in patients after TBI. We assume that these test results have probably influenced the “physicians’ diagnosis”.

At follow-up after a median time of seven months after injury, the prevalence of lowered IGF-I values indicative of somatotropic insufficiency and the prevalence of lowered sex hormones indicative of gonadotropic insufficiency decreased. The decrease was predominantly observed when Criterion 1 (basal laboratory values) was applied, but it was also present when Criterion 2 (basal laboratory values/hormone replacement therapy) or Criterion 3 (physicians’ diagnosis) were used as the definition for pituitary insufficiency. It should be kept in mind that severe critical illness can be considered as chronic stress. In stress physiology, hypercortisolism is observed as stress response to prolonged stress in critical illness [18,19]. However, hypercortisolism itself leads to a downregulation of gonadotropin levels [20]. In patients recovering from their brain injury, chronic stress might be reduced over time, which might provide a normalization of the corticotropic and gonadotropic axes [21]. This is an important fact, because persistent hypogonadism six and 12 months after injury is associated with worse functional and cognitive outcome in patients after TBI, as described in the study of Wagner *et al.* [22].

In clinical routine, it is important for the treating physician to know if a re-testing of patients after TBI or SAH should be recommended or not. In order to answer this question, we looked at intraindividual changes (*i.e.*, new onset insufficiency or recovery from hypopituitarism). In general, most patients showed similar results in the first hormonal assessment compared to the follow-up. In more than 77% of all cases, the results (insufficiency or no insufficiency) remained at follow-up. Changes in the individual patient were predominantly observed in the somatotropic, gonadotropic and corticotropic axes. Especially in patients after TBI, IGF-I values at first hormonal assessment often returned to normal at follow-up. These facts have to be considered in the consulting and treatment of patients after TBI and SAH.

The pathomechanisms of secondary worsening of pituitary function months to years after the injury are still largely unresolved. A few studies provide preliminary evidence that injury-induced hypothalamic-pituitary autoimmunity might play a role [23,24]. In analogy to the scarce data on delayed hormonal dysfunction in patients after radiotherapy, it can also be postulated that hypothalamic neurotransmitter pathways could be affected by the injury [25]. Last, but not least, it is also conceivable that hormone secretion capacity decreases over time to gliotic scarring or secondary neuronal degeneration as a consequence of the initial injury to the pituitary. Further research into this matter is clearly needed.

The strength of our study is that we could investigate a large cohort of 168 patients with different diagnostic criteria (laboratory values, need for a hormone replacement therapy and physicians’ diagnosis) in a multi-center study. Our study has a naturalistic design to be close to clinical routine. Furthermore, we do not only show cross-sectional data on the hormonal situation of brain-damaged patients, but we were also able to present longitudinal data of a large number of patients.

Nevertheless, our study has also several limitations. First, a follow-up was not generally mandatory in all of the 1242 patients originally included into the Structured Data Assessment of Hypopituitarism. This means that there might be a preselection of patients from the total cohort [26] who received a follow-up testing, e.g., severely impaired patients or patients with borderline laboratory values or clinical changes over time. These were patients who were still on treatment after TBI and SAH and consulted their physicians. Furthermore, we did not have a predefined time of re-testing for these patients. In addition, we did not report on stimulation tests, which can help to diagnose especially corticotropic and somatotropic insufficiencies in a better way. In the third place, testosterone replacement therapy was not documented in most of the male patients. Therefore, we could not specify if the patient has a gonadotropic insufficiency despite normal laboratory values. Hence, our results on the gonadotropic insufficiency in men according to lowered basal laboratory values cannot be directly compared to the other pituitary axes. As the basal laboratory values were not evaluated in a central laboratory, we cannot demonstrate mean pituitary hormone levels *per se* due to different cut-off levels and different test methods. We classified laboratory values according to the reference values of each center. In some pituitary hormones, an age-dependent decline can be observed. However, age-dependent reference values in adults had only been applied for IGF-I, not for testosterone or other hormones. Finally, we were not able to include a neuropsychiatric evaluation of our patients, although it would have been of interest to correlate neuroendocrine disturbances with neuropsychiatric impairment.

Overall, we could show that hypopituitarism after TBI and SAH remains predominantly stable. The conclusion that can be drawn from our study is that patients should be informed about the high probability of chronic posttraumatic hypopituitarism. Nevertheless, there is a potential recovery from gonadotropic and somatotropic insufficiencies, especially in patients with lowered IGF-I levels after TBI. These facts should be considered treating patients after TBI and SAH.

## 4. Materials and Methods

### 4.1. Structured Data Assessment of Hypopituitarism

Neurological, neurosurgical and endocrinological centers in Germany (*n* = 13) and Austria (*n* = 1) participated from 2005 to 2008 in the Structured Data Assessment of Hypopituitarism after TBI and SAH. Data from 1242 patients were collected using a structured study sheet involving information on clinical, radiological and hormonal parameters, as well as sociodemographic values. These data were collected by the treating physicians in the specialized centers. It was allowed to include patients retrospectively or to evaluate hormonal status prospectively during the study period up until 2008. Patients were entered into the Microsoft Access^®^-database management system online after pseudonymization. All patients or their legal representatives gave written informed consent. The study was approved by the ethics committee of the Bavarian State Chamber of Physicians in Munich with date of 21 April 2005, and complied with the Declaration of Helsinki.

Details on the methodology were described previously by Kreitschmann-Andermahr *et al.* [27]. The results of the whole cohort were presented previously by Schneider *et al.* [26], and data on the long-term insufficiencies after TBI and SAH were described by Krewer *et al.* [28].

### 4.2. Subject Characteristics

Subject characteristics are displayed in Table 3. The severity of disability in patients after brain damage is indicated by the Glasgow Outcome Scale [29] with the following graduation: 1, death; 2, persistent vegetative state; 3, severe disability (conscious, but disabled); 4, moderate disability (disabled, but independent); and 5, good recovery.

**Table 3:** Subject characteristics.

Type of Injury	All	TBI	SAH	*p*-Value ^◊^
**Number of Patients**	*n* = 168	*n* = 81	*n* = 87	-
Sex (male/female)	87/81	57/24	30/57	<0.0004
Age * mean ± S.D. (years)	43 ± 15	40 ± 18	46 ± 10	0.013
Age ^#^ mean ± S.D. (years)	45 ± 14	43 ± 17	46 ± 10	0.103
Age ^#^ median (25th, 75th percentile) (years)	45 (38, 55)	43 (27, 56)	47 (40, 52)	-
GOS median (25th, 75th percentile)	5 (4, 5)	5 (4, 5)	4 (4, 5)	0.559
BMI mean ± S.D. (kg/m^2^)	26 ± 5	26 ± 6	26 ± 4	0.87
1st test ^**†**^ median (25th, 75th percentile) (months)	3 (1, 16)	3 (1, 40)	3 (2, 9)	0.734
Δ 2nd to 1st test median (25th, 75th percentile) (months)	7 (2, 11)	4 (1, 8)	8 (5, 15)	<0.0004

Abbreviations: BMI, body mass index; GOS, Glasgow Outcome Scale; * at injury; ^#^ age at first hormonal evaluation; ^**†**^ time between injury and first hormonal assessment; ^**◊**^ *p*-value to compare patients after TBI and SAH.

### 4.3. Hormonal Assessment

Hormone levels were measured in the local laboratories of the participating centers and recorded in the study sheets. Reference values differed from center to center in dependence of the specific assay applied and on the local laboratory value. The following definitions of hormone deficiencies were used according to the local laboratory reference range:

Corticotropic axis: basal cortisol below the local laboratory reference value, e.g., <62 µg/L, <5 µg/dL or <171 nmol/L, respectively;

Gonadotropic axis in men: basal testosterone below or above the local laboratory reference value, e.g., <9.9 and >27.8 nmol/L or <2.8 and >8 µg/L;

Gonadotropic axis in women: estradiol in women below the local laboratory value (e.g., proliferative stage < 13 ng/L or < 18.9 pg/mL, respectively, ovulation < 86 ng/L or < 35.5 pg/mL, respectively, luteal phase < 44 ng/L or < 22.4 pg/mL, respectively, postmenopause < 35 ng/L or < 10 pg/mL, respectively, depending on the local laboratory); basal laboratory values in female patients with unknown menstruation status (premenopausal or not) were excluded. If laboratory values could not be adjusted to the menstruation cycle phase, the reference value was not clear, and the data were therefore excluded from further analyses.

Thyrotropic axis: free thyroxine (fT4) below the local laboratory value, e.g., <12 pmol/L or <0.93 ng/dL; 

Somatotropic axis: IGF-1 level below the local age- and sex-specific reference value;

Lactotropic axis: prolactin level below the local sex-specific reference values.

Although it was recommended to investigate all pituitary axes, endocrine assessment was in some cases incomplete. In total, *n* = 385 study sheets (168 study sheets from the first time of testing and 217 follow-up study sheets) were available. In *n* = 15 female patients, estradiol levels could not be adjusted to the reference values, since the menstruation cycle phase was unknown. In *n* = 23 female patients, estradiol values could not be used, since it was not assessed if they were premenopausal, perimenopausal or postmenopausal. After quality control, 327 cortisol levels, 329 fT4 levels, 151 testosterone levels (out of 193), 108 estradiol levels (out of 192), 309 prolactin levels and 313 IGF-I levels remained for further analyses.

In *n* = 46 patients, a hormone replacement therapy has been established after the diagnosis of posttraumatic hypopituitarism. *n* = 101/385 study sheets and the therein included laboratory values have been measured on hormone replacement therapy. In *n* = 242 study sheets, a hormone replacement therapy due to posttraumatic hypopituitarism was denied. In *n* = 42 patients, data regarding a hormone replacement therapy were missing.

### 4.4. Statistical Analyses

The Structured Data Assessment of Hypopituitarism after TBI or SAH database and data administration were realized by ANFOMED GmbH (Möhrendorf, Germany).

Data are displayed as the mean ± standard deviation if normally distributed and, otherwise, as the median and the 25th and 75th percentiles. Differences in sex between the subgroups regarding the subjects’ characteristics were analyzed using a χ^2^ test. To test for significant differences between patients after TBI and SAH, age at injury and age at first testing were analyzed using the two-tailed Student’s *t*-test. The Glasgow Outcome Scale (GOS), time at first hormonal assessment and time interval between first and second hormonal assessment were analyzed using the Mann–Whitney *U*-test.

Each patient was classified as: (1) showing no change in hormonal status over time; (2) developing a new insufficiency over time; or (3) recovered from hypopituitarism over time. Based on this classification, a binary logistic regression analysis was performed for several pituitary axes. Forward stepwise regression was used, in which variables are added to the model in the order of their statistical significance. The variables considered influential included “age at injury”, “sex”, “time between injury and first testing”, “type of injury” and “pituitary insufficiency at first visit”. All statistical analyses were performed with IBM SPSS Statistics 22 (IBM Corporation, Armonk, NY, USA).

## 5. Conclusions

In our study, we could show that hypopituitarism after TBI and SAH remains predominantly stable. Patients should be informed about the high probability of chronic posttraumatic hypopituitarism. Nevertheless, there is a potential recovery from gonadotropic and somatotropic insufficiencies, especially in patients with lowered IGF-I levels after TBI. These facts should be considered treating patients after TBI and SAH.

## Supplementary Materials

Supplementary materials can be found at http://www.mdpi.com/1422-0067/17/1/2/s1.
