# Supplementary material for: The Development of Neuroendocrine Disturbances over Time: Longitudinal Findings in Patients after Traumatic Brain Injury and Subarachnoid Hemorrhage
Source: Int J Mol Sci. 2015 Dec 22;17(1):2. doi: 10.3390/ijms17010002 (PMC4730249; doi:10.3390/ijms17010002)
Supplement: Supplementary file 1 [file ijms-17-00002-s001.pdf]

Supplementary Materials: The Development of Neuroendocrine Disturbances over Time: Longitudinal Findings in Patients after Traumatic Brain Injury and Subarachnoid Hemorrhage

Anna Kopczak, Carmen Krewer, Manfred Schneider, Ilonka Kreitschmann-Andermahr, Harald Jörn Schneider and Günter Karl Stalla

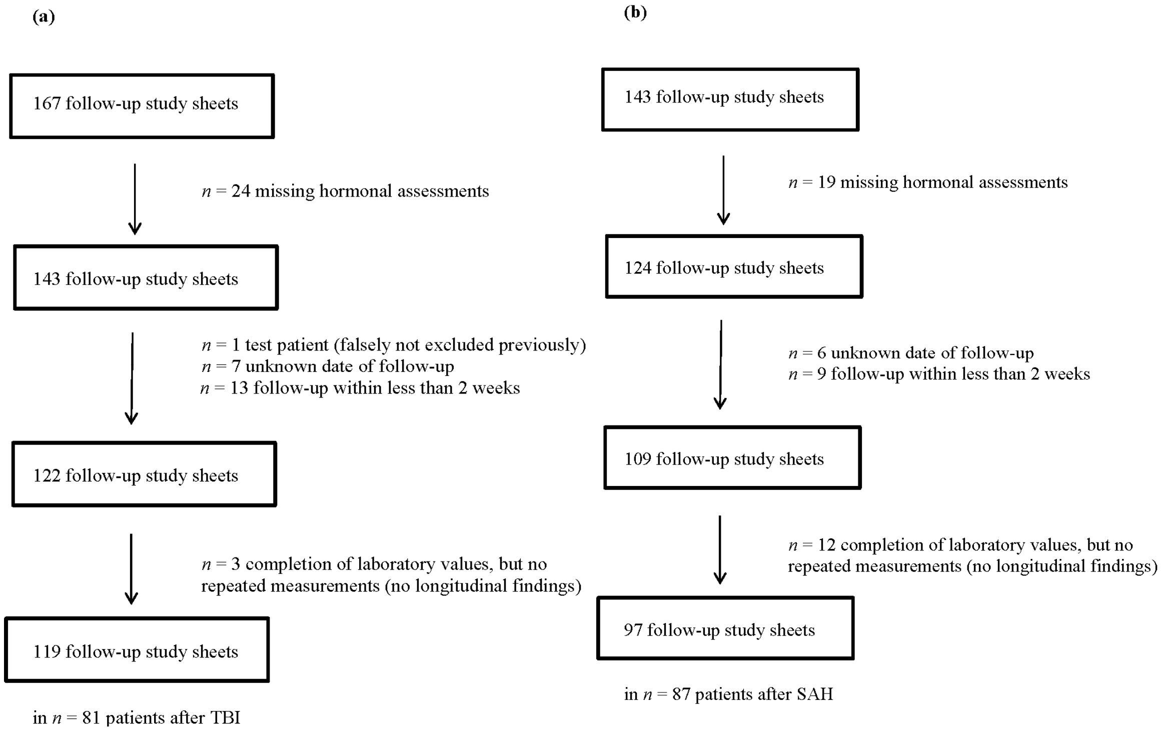

Figure S1. Follow-up study sheets in patients after traumatic brain injury (TBI) (**a**) and after subarachnoid hemorrhage (SAH); (**b**) included in the Structured Data Assessment of Hypopituitarism.
